# Swarm Smart Meta-Estimator for 2D/2D Heterostructure Design

**DOI:** 10.1021/acs.jcim.3c01509

**Published:** 2023-10-05

**Authors:** Romain Botella, Andrey A. Kistanov, Wei Cao

**Affiliations:** Nano and Molecular Systems Research Unit, Faculty of Science, University of Oulu, FIN 90014 Oulu, Finland

## Abstract

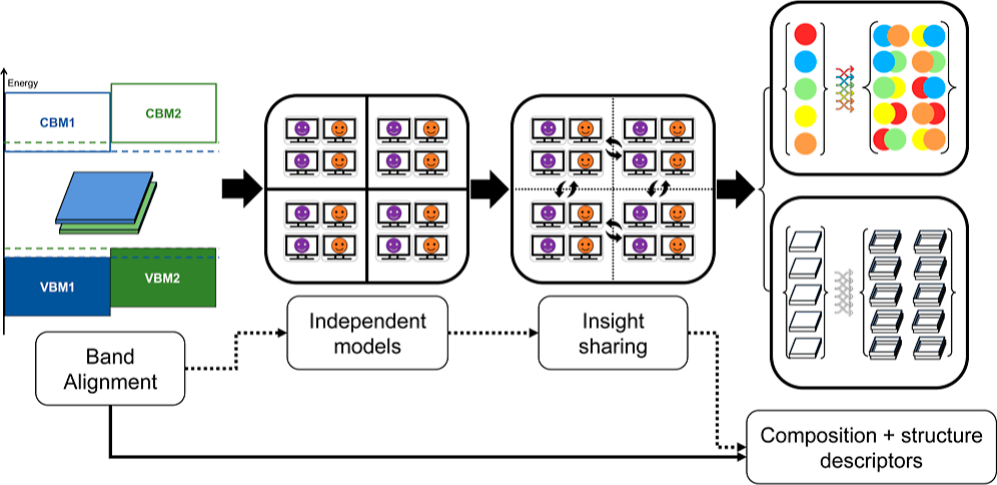

Two-dimensional (2D) semiconductors are central to many scientific fields. The combination of two semiconductors (heterostructure) is a good way to lift many technological deadlocks. Although ab initio calculations are useful to study physical properties of these composites, their application is limited to few heterostructure samples. Herein, we use machine learning to predict key characteristics of 2D materials to select relevant candidates for heterostructure building. First, a label space is created with engineered labels relating to atomic charge and ion spatial distribution. Then, a meta-estimator is designed to predict label values of heterostructure samples having a defined band alignment (descriptor). To this end, independently trained k-nearest neighbors (KNN) regression models are combined to boost the regression. Then, swarm intelligence principles are used, along with the boosted estimator’s results, to further refine the regression. This new “swarm smart” algorithm is a powerful and versatile tool to select, among experimentally existing, computationally studied, and not yet discovered van der Waals heterostructures, the most likely candidate materials to face the scientific challenges ahead.

## Introduction

Semiconductors are key materials to various technologies such as photocatalysis, electronics, and energy harvesting. The chemical space of semiconductors is spreading over many compositions and many structures, but also several morphologies.^1,2^ Among them, two-dimensional (2D) materials, consisting of a monolayer held together by strong (ionocovalent) interactions, present many interesting properties such as high surface area, high exciton binding energies due to modified dielectric function in confined environment,^3−6^ mechanical stiffness and flexibility enabling gap engineering,^7^ and magnetic properties with their high potential for spin–orbit-coupling.^8,9^

The combination of two semiconductors (heterostructures) bolsters the applicative power of such materials even more by enabling them to compensate for the shortcomings from one material (given a specific application, e.g., a high recombination rate for photocatalysis) with the advantages of another [e.g., more suitable conduction band minimum (CBM)/valence band maximum (VBM)].^10^ One central characteristic of the heterostructure is the band alignment, constituted by the difference in CBM (CBMDiff) and the difference in VBM (VBMDiff). This quantity is key to know the direction and strength of the charge transfer.^10^ Most of the 2D/2D heterostructures are called van der Waals (vdW) heterostructures, presenting low charge transfer between layers^11^ and increasing the amount of prospective combinations.^12^ They find applications in many fields, such as photocatalysis,^13^ electronics,^14^ and lubrication.^15^

Brute force methodologies, whether experimental or computational, in order to find the best 2D/2D heterostructure candidate for a given application require significant labor and time input,^16^ with no insurance as to the outcome. For example, first-principles calculations are particularly useful for simulating small sets of compounds with high accuracy.^17^ To lift this deadlock of computational demand, machine learning (ML) approaches are used.^18,19^ These methods empower research to perform tasks ranging from predicting material properties such as adsorption energy and formation energy,^20^ p*K*_a_ of macromolecules,^21^ or chromatic dispersion of compounds,^22^ to simulating structures with high accuracy using deep neural networks.^23^ The cost/accuracy trade-off can increase in sophistication by using ML to decrease the computational effort of such methods,^24,25^ or by recommending compounds (molecules, materials) on which to apply high accuracy, computationally demanding methods.

While the success of deep neural networks is well-known,^26,27^ ensemble learning methodologies consisting of several base models combined in a meta-model (meta-estimator or meta-classifier for regression or classification purposes, respectively) to improve predictive power have been successful as well.^28^ Among these models, random forest (RF) is the most used because of its high applicability and interpretability.^29,30^ On a similar idea of strength in numbers, particle swarm optimization has also received attention from the research community.^31,32^ It is based on swarm intelligence principles: several predictive entities (particles) can interact with each other to improve predictive power. Each entity has its own answer to the question [cognitive learning (CL)], and the concerted answer [social learning (SL)] is an improvement of the isolated answers.

With regards to vdW heterostructures, studies have been focusing on straightforward combinations, e.g., Ge/Si^33^ or graphene/borophene,^34^ to predict their properties with both high speed and accuracy. Other works focused on the prediction of properties for different types of heterojunctions by choosing several well-known 2D materials, losing in generalization power.^25^ Sampling a large part of the material space following Anderson’s rule for band alignment has also been used in previous work.^35^ Another model has been trained to determine the heterojunction type (I, II, or III) obtained by combination of materials.^36^ The descriptors used are numerous, comprising the energy branch point, known to have a good correlation in heterostructure properties,^36−38^ property-labeled materials fragments descriptors^15^ as well as first-principles calculated properties. Although many powerful indicators have been used to predict various properties, new descriptors can still prove useful in reducing the dimensionality of the data set and improve generalizability. Furthermore, instead of predicting properties based on already known materials, a more challenging task is to find and investigate new composites with targeted properties.

Moreover, most of ML works are focusing on predicting properties of materials one by one (for this material, what is the value of the targeted properties?) rather than looking for potential candidate with a defined research target (for this research target, e.g., visible light absorption, what kind of materials should I look for?). For the former scheme, label values are the research targets, and it is well suited for having a better knowledge of materials already accessible or considered.^18^ On the other hand, for the latter scheme, the labels are material characteristics, and they are more adequate for finding new candidate materials (not yet accessible nor considered) suiting specific needs (the actual research target). By interchanging the nature of the labels and the descriptors, the prediction scheme is entirely modified, allowing for new discoveries.

The present work aims at tackling such a task. By creating general and accessible labels that are characteristic of any vdW heterostructure, the distribution mismatch (DM) and the charge mismatch (CM), a 2D label space is created. In it, up to 79,003 2D/2D heterostructures created from randomly picked 2D materials are displayed. This enables efficient and interpretable search of the material space to quickly test materials for target properties by predicting their most basic characteristics (partial charge of atomic constituents and their spatial distribution). The aforementioned labels being independent of any structural or compositional considerations, all heterostructures can be compared and a large data set, representative of the material space targeted, has been collected. From the large size of the data set, the heterostructure space can be explored using a combination of swarm intelligence and ensemble (boosting) methods improving low level, easily interpretable base estimators. This is applied for the first time to the heterostructure search herein. We also use another prediction scheme where we start from what is expected (defined research targets, e.g., defined band alignment) to obtain label values in line with expectations (sample characteristics suiting the actual research target). This prediction scheme is breaking with the usual trend of ML works (from one material to its properties) mentioned earlier.^15,18,25,35,36,38^ Heterostructures from the literature, similar to those that the meta-estimator can predict, are shown, highlighting the relevance of the model to the field.

## Methods

### Data Collection and Validation

ML approaches are data-driven. As such, their success relies on the large and diverse amount of data treated to make predictions and the way the data set is constituted. High-throughput calculations provide pools of compounds computationally obtained, which is of great use for data-driven research works. Those materials are either used in experimental work or are waiting to find an application.^39−41^

From the 2DMatPedia database, randomly picked 2D materials were used to calculate the VBM, CBM, and vacuum levels needed for band alignment calculations. The 2DMatPedia database is entirely available as a JSON file. The samples were straightforwardly picked using a random number generator and picking the atomic position file and conditions of the material associated with the index number picked.

For the chosen materials, spin-polarized first-principles calculations were performed by using the plane-wave Vienna ab initio simulation package (VASP).^42^ The Perdew–Burke–Ernzerhof functional (PBE) exchange–correlation functional under the generalized gradient approximation^43^ together with the vdW corrected functional were implemented.^44^ The optimized structures of 2D materials were taken from the 2DMatPedia database and used to conduct the electronic relaxation step of the calculations. Descriptors and labels were extracted from VASP output files produced by these calculations.

Structural and electronic parameters were then extracted from VASP outputs to enable heterostructure-related descriptor/label value calculations. The structural information was extracted from atomic positions, and electronic parameters (charges) were obtained through Bader analysis. Heterostructure samples were produced applying Anderson’s rule.^11^ This assumption has been previously used to predict heterostructure properties with ML approaches.^35^ From 410 2D materials that were taken at random and reproduced from the database, 79,003 heterostructures were created.

To avoid any convention issues, all values were made positive. As a consequence, the order of semiconductors 1 and 2 (assuming a 2 semiconductors heterostructure) is not fixed. This lifts potential constraints that the material order could exert on the prediction outputs.

### Base Model Training and Cross-Validation Procedure

Before training of the models, all data were standardized ∼ *N*(μ,σ) to provide better prediction results. The predictions are transformed back to their initial values to compute the scoring function values. K nearest neighbors (KNN) regression was chosen as the base estimator for the predictions, and the justification of this choice can be found in the Results section. This is a nongeneralizing, nonparametric model that interpolates data points to produce the prediction results. KNN algorithms are generally used for classification purposes.^29,45,46^ To study the performance of the base model, the mean absolute deviation (MAD) is calculated for the test set. The data set was conventionally split into a training and a test set at a 80:20 ratio (63,202 elements for training and 15,801 elements for testing). A 20-fold cross-validation was performed on the data set, chosen from a screening of *k* values. All the results shown herein come from generalization tests following the same protocol of clearly separating training and test sets. Algorithm parameters were optimized on a reduced data set for computational demand reasons. The best MAD scores were obtained with 10 neighbors search, Euclidean distance as a metric, and using distance-weighted interpolation. The neighbor search algorithm was automatically determined by the machine to reach the lowest computational costs. ML endeavor work was performed using the scikit-learn library.^47^ Pandas,^48^ Numpy,^49,50^ and Matplotlib^51^ libraries were used to respectively display, manipulate, and visualize the data.

### Labels

Two labels have been engineered to represent heterostructured samples. They are defined in this section and explained in the Results section. One of them focuses on the spatial distribution of atoms in the material; the other is more focused on the charges of ions in the constituents of the heterostructure. For materials 1 and 2, the DM is defined as the difference between the variances of the positions of the atoms in their unit cells
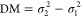
1where σ_2_^2^ and σ_1_^2^ are variances calculated for 2D materials (*x* and *y* positions). This label is configuration-independent, as it can be shown that it does not change upon exchanging the centers of unit cells 1 and 2.

**Figure 1:**
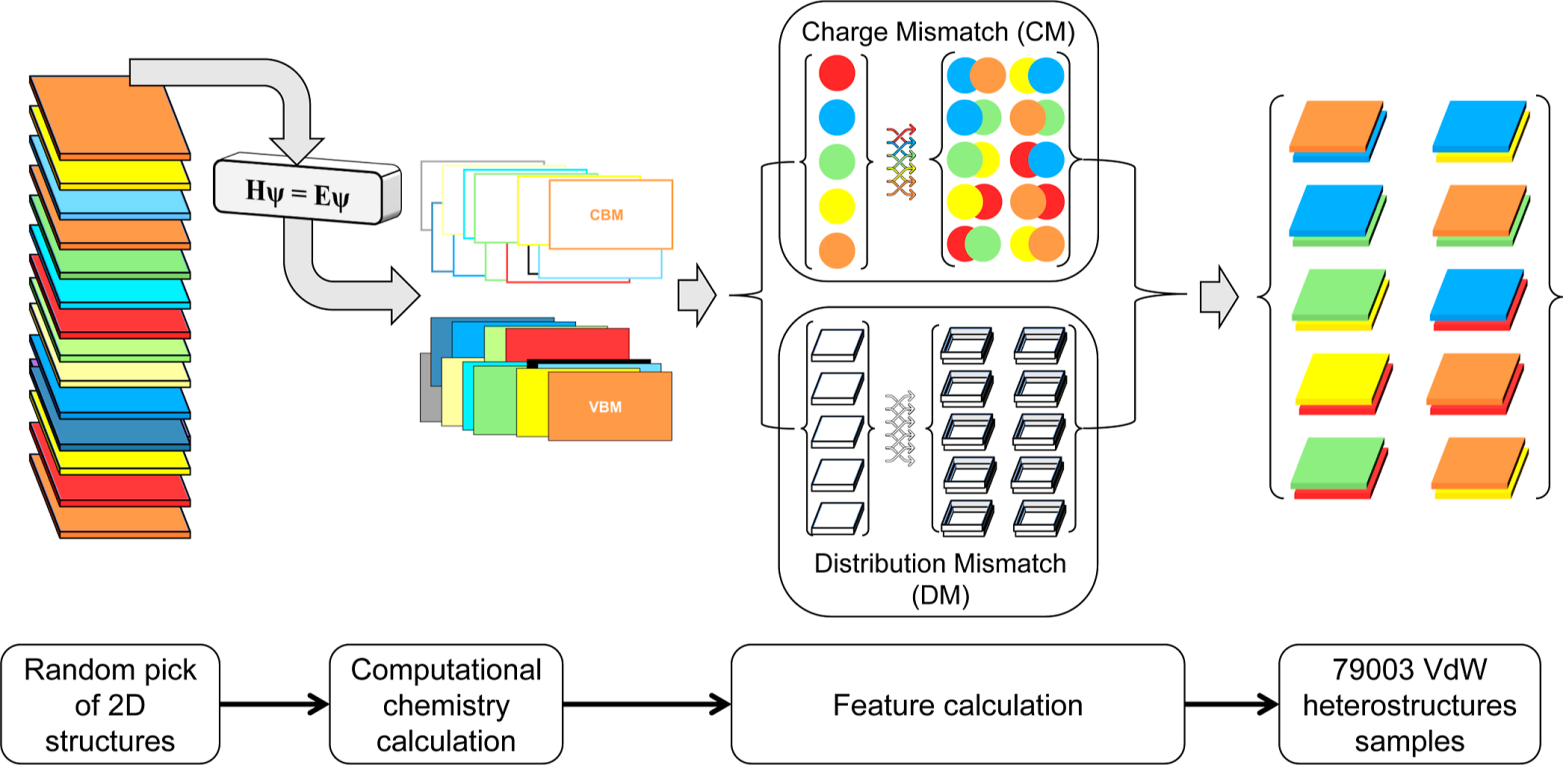
Diagram depicting how the data were extracted from first-principles calculations, separated in labels, and then combined to make the heterostructure space.

The CM is the compositional label. It arises from the simplification of the physical truth to be generalizable to many different materials (details in the Results section). The CM is defined as
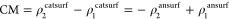
2with ρ_*i*_^catsurf^ the surface density of cations in material *i*, ρ_*i*_^ansurf^ the surface density of anions in material *i* (*i* = 1, 2). These two labels constitute the label space, in which all heterostructures can be represented, regardless of structure or composition, with a specific (CM, DM) pair. The separation of electronic and structural labels from 2D materials calculated from first-principles is depicted in Figure 1.

## Results

### Label Space Engineering

Prior to model training, a label space is created by designing labels associated with the whole data set. These labels must reflect the characteristics of the object studied.

The most used parameter to classify and distinguish heterostructures is the lattice mismatch (LM). It is known that this quantity influences the charge carrier transport throughout the heterojunction and is paramount for the epitaxial growth of heterostructures.^52,53^ An important LM is going to apply a strain on the material, which can impair charge transfer further as well as modify the band structure.^54,55^ This is especially known in first-principles studies of heterojunctions.^56,57^ LM is mainly relevant for unit cells with the same symmetry, as a change of crystal structure can change the shape of the unit cell. This can, in turn, impair the possibilities to simulate such combinations of solids, despite their prospective applications in experiments. It also decreases the relevance of comparing only the length of lattice vectors, which is not the only parameter changing from one crystal structure to another. Moreover, the definition of a lattice neglects the positions of the different atoms in it and their distribution (base), forming the crystal structure.^58,59^

To reduce the dimensionality of the label space, as well as addressing some lacks of LM heterostructure characteristics, the crystal structure (lattice + base) is described as a distribution of atoms in space (base) herein, ignoring the symmetry considerations underlying their positions. They are, however, implicitly included in this label, as is the LM.

From this model, two quantities characterizing the distribution of positions are the mean position (*r̅*) and the variance (σ^2^). The mean position of the atoms in a unit cell corresponds to its center and presents little interest, while the variance indicates the dispersion of the atoms around the center, which can change from one material to another. To the best of our knowledge, this has never been used before in related literature. We use DM (defined in Methods) in place of LM for this work.

Regarding composition, focusing on the interface between two materials constituting the heterostructure, the charge transfer can be approximated by a Coulombian interaction of the charge carrier with the field produced by the two materials with different charge densities (because of different compositions/bonding environments).^60^ In this approximation, a charge carrier located at equidistance from two semiconductors will tend to go wherever the potential is lower, i.e., on average, toward the material with the highest density of charge from the opposite sign. In equation, this translates to
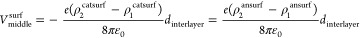
3Hence
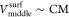
4considering a negative charge localized at equidistance of materials 1 and 2, and with *d*_interlayer_ the interlayer distance between the two materials, ρ_*i*_^catsurf^ the surface density of cations in material *i*, ρ_*i*_^ansurf^ the surface density of anions in material *i*, e the elementary charge, ε_0_ the vacuum permittivity, and CM the charge mismatch (defined in Methods). According to this quantity, an electric charge will be more attracted to the most charged surface, and the interaction will be stronger, the higher CM is. Moreover, these two labels allow any structure to be compared without any additional calculations, using atomic positions obtained in databases or from XRD measurements. The cell parameters can also be found the same way, and the charge of considered structures can be straightforwardly computed from the application of the electroneutrality principle. These labels are only based on ionic positions and charges that are common to any composition and any structure group. The values of CBM difference (CBMDiff), VBM difference (VBMDiff), CM, and DM constitute the data set.

### Data Set Overview

**Figure 2:**
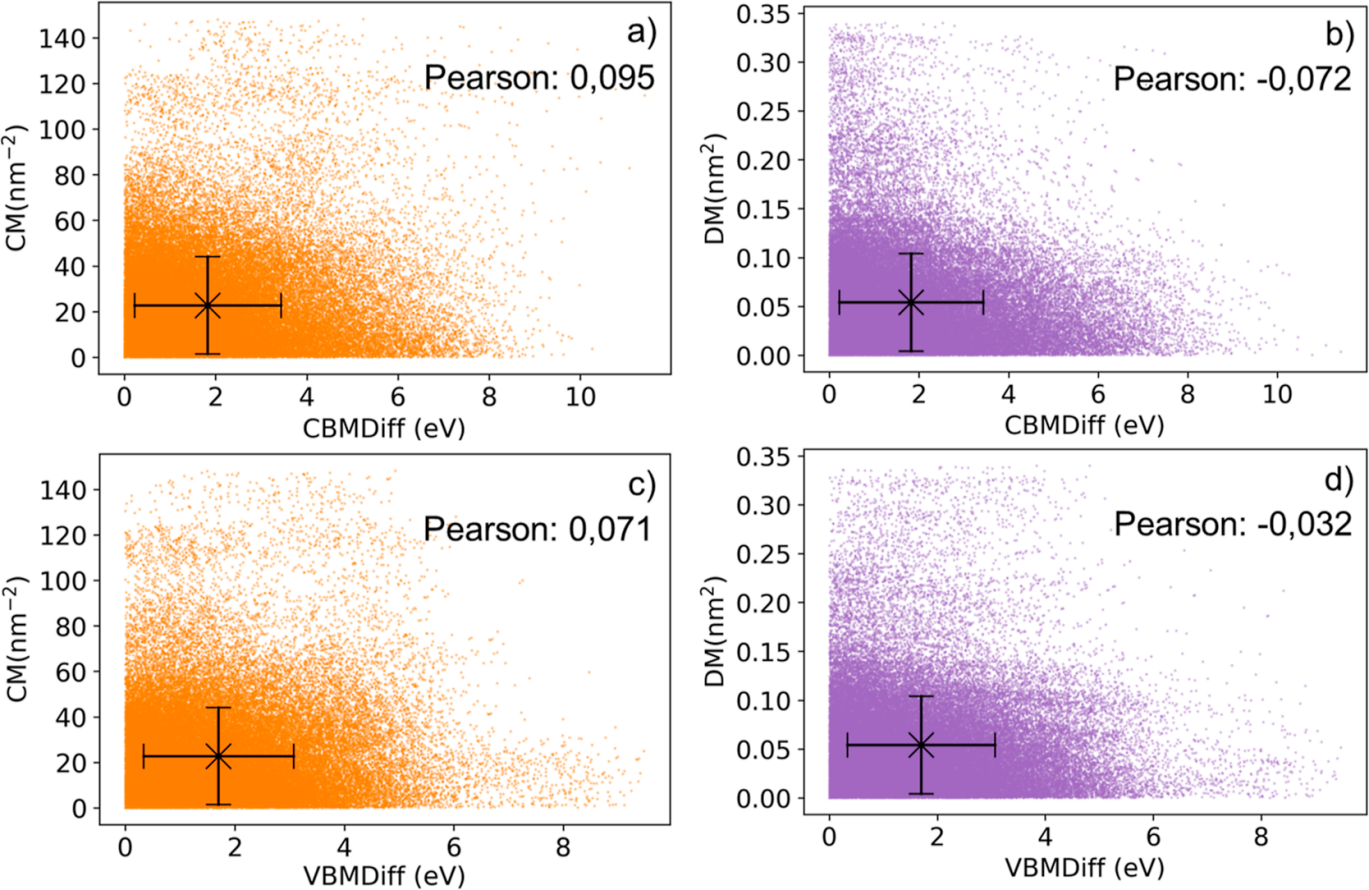
(a) CM as a function of CBM difference (CBMDiff), (b) DM as a function of CBMDiff, (c) CM as a function of VBM difference (VBMDiff), and (d) DM as a function of VBMDiff. For each graph, the Pearson correlation coefficient is given.

One of the most important parameters for heterojunction design is the band alignment, giving an indication on the strength of the charge transfer between the two semiconductors. It is composed of a CBM difference (CBMDiff) and a VBM difference (VBMDiff). The full data set (training and testing sets) is shown in Figure 2 in four graphs presenting the correlation of all descriptors with the different labels, as the formers are going to be used to predict the latters. While including the entire 2D/2D heterostructure space is impossible, randomizing the selection of heterostructure candidates for the data set has two main advantages: it ensures a good sampling of the material subspace targeted and enhances the generalizability of the model.

As seen in Figure 2, the graphs exhibit a lack of trend, consistent with low correlation between the targets and the descriptors, as confirmed by Pearson coefficient values. The distribution of the descriptor and label values in Figure 2a–d is centered at (1.1828 ± 1.6072 eV, 22.7164 ± 21.31 nm^–2^), (1.1828 ± 1.6072 eV, 0.0541 ± 0.05 nm^2^), (1.7057 ± 1.3686 eV, 22.7164 ± 21.31 nm^–2^), and (1.7057 ± 1.3686 eV, 0.0541 ± 0.05 nm^2^), respectively. They are mainly determined by the values of the DM and CM labels. Standard deviations of the different label and descriptor values are delimiting the highest density regions of the data set. As the density of the data set in the label space fades for CM > 43 nm^–2^ and DM > 0.10 nm^2^, sampled compounds start to show quite high values. These values can come from sampled compounds diverging from the ones we aim to select, having high thickness, or many elements incorporated in the structure. The high density of the data set suggests a good sampling of all existing 2D/2D heterostructures.

### Choice and Performance of the KNN Base Model

There is a necessity for a precise value of band alignment for the use of materials in several applications.^13^ This justifies that these two quantities (VBMDiff and CBMDiff) are defined as inputs (traditional research targets, descriptors in this work) for the present model. CM and DM are the outputs (label values corresponding to the research targets in this work).

Regarding the choice of base model to be used in the regression, both the high density and the lack of structure observed in Figure 2 motivated the use of a nongeneralizing, nonparametric model such as KNN regression that will adapt to the data set to predict label values in a straightforward manner (interpolation). This strong interpretability at the core of the architecture is then transmitted to the rest of the meta-estimator. The KNN algorithm has advantages such as high interpretability, diversity of uses, and reasonable accuracy, as well as drawbacks, among which is the need to optimize different parameters (e.g., number of neighbors and metric) for model training as well as high memory cost need due to its laziness.^62^ While the drawbacks of this algorithm can be mitigated by thorough optimization of algorithm parameters as well as search algorithm changes (see Methods section), the advantages are kept, making this algorithm a suitable choice for the high density exhibited by the as-created data set.

**Figure 3:**
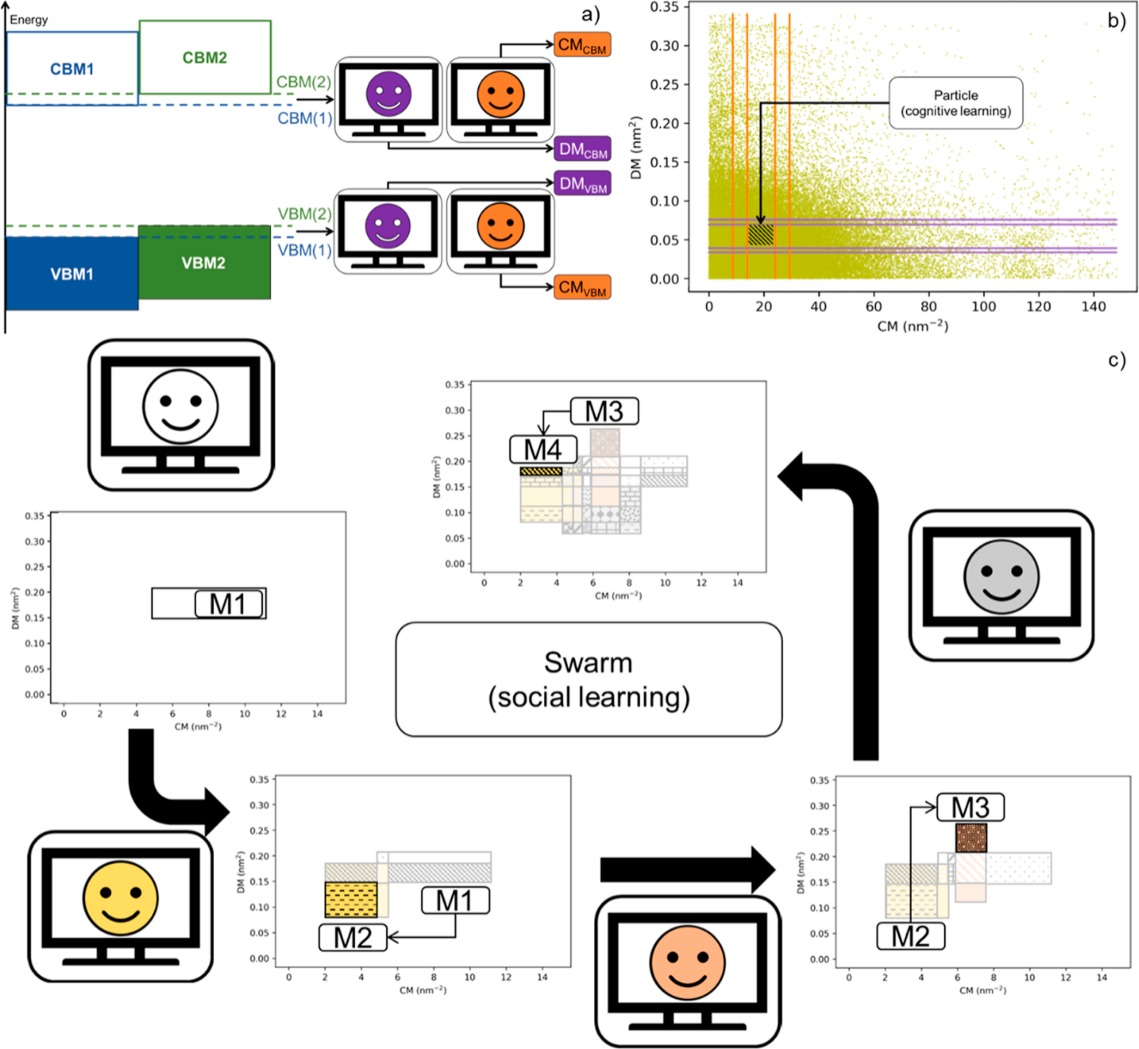
(a) Diagram illustrating the particle formation by KNN regressor boosting from descriptors (band alignment) to labels (CM and DM), (b) label space and particle, representation of CL (the size of the particle is indicative), (c) swarm formation, representation of SL by secondary particle formation. For (b), the vertical orange lines correspond to the ranges of CM values predicted, and the horizontal purple lines correspond to the range of DM values predicted. M1, M2, M3, and M4 are highlighting maximum target density regions, changing with increasing swarm size.

After training of the KNN regressors, the MAD obtained after the 20-fold cross-validation procedure was around 12% of the entire range of label values. A study of the learning curve showed no significant improvement of the MAD upon increasing the size of the training set, indicating the base KNN models used on the data set as weak learners. One important advantage of the weak learner is that a reduced batch size can be used. From the learning curves obtained, a 900-sample batch size has been chosen for the generalization tests to ensure the stability of the MAD score, alleviating the high memory cost of the KNN algorithm. The use of *k*-fold cross-validation implies a clear separation of the testing and training set, preventing any overfitting of the data. This high accuracy comes from the high density of the data set acquired and the adequate choice of base model. However, it is not able to single out one data point (which could be misinterpreted for an overfitting) but a larger group of compounds (Figure 3b). This observation is suggesting a good balance between overfitting and underfitting as needed for any high-quality ML work.^63^ While the results of KNN prediction are acceptable, they can still be further improved by combining several predictions in an ensemble method.

From this point on, any result shown herein is a generalization result, i.e., a result obtained through testing for band alignment values that do not correspond to any material in the data set.

### Swarm Smart Meta-Estimator Architecture

The label space contains two elements, CM and DM. Those are the outputs of the different KNN models, obtained from the band alignment values given as inputs. It enables to represent all fabricated heterostructures, regardless of crystal structure or composition. To single out one subset in a two-dimensional label space, two independent KNN regressors are trained (Figure 3a), the overlap of their regression results in CM and DM defines a subset in the label space (Figure 3b). The choice of KNN regressor as the base model is explained in the previous part. Useful results on the given data set are obtained via a mixture of different regression strategies. To improve the base model chosen (KNN), a boosting strategy is used by training two of them separately on different labels, one on CM and one on DM. Boosting strategies are part of ensemble methods,^28^ enabling to increase predictive power through the interaction of different algorithms. The label space particles are defined using the MAD on the prediction of label values and centered on the predicted value. This first subset is assigned to a CBMDiff or VBMDiff target. To have both CBMDiff and VBMDiff taken into account, the same boosting strategy is repeated once more to constitute a second subset, this time associated with the other target not yet predicted (CBMDiff or VBMDiff, depending on the descriptor assigned to the first subset). As the main goal of the model is to find heterostructures with label values associated with a given total band alignment (given a (CBMDiff, VBMDiff) pair), the label intervals associated with the band alignment targets are defined by the overlap of the two subsets defined above, i.e., by the overlap of the 4 KNN regressions (KNN boosting, Figure 3a,b), forming a particle in the label space. Then, to increase the precision of the KNN boosting method, swarm intelligence principles are used by performing SL on the different regression results from the KNN boosting, which is a new use of swarm intelligence, usually based on points and not on whole regions of the label space.

By sequentially building particles (based on boosted KNN regressors), a particle swarm is formed. The swarm is composed of particles at different locations in the label space and with different sizes (forming a swarm pattern), as represented in Figure 3c. According to swarm intelligence principles,^31,32^ the learning process of the model is 2-fold: CL first, corresponding to the prediction of the particle independently formed, and the SL corresponding to the prediction of the secondary particle formed after interparticle interaction (information sharing). In this work, the CL comes from the KNN boosting (Figure 3a), and the SL consists in the mixing of the bounds of the different initial particles (assumed equivalent), resulting in the formation of secondary particles. These secondary particles come from the interparticle sharing of information about areas containing targeted heterostructure samples (Figure 3b). The bounds of the secondary particles, made from SL, constitute an ensemble of regression results. To evaluate the performance of the regression as well as to choose the values associated with the desired heterostructures, a set of scoring functions is designed based on the content of the particles obtained by the regression. The first score is the CL score, measuring the percentage of the target heterostructures that the particle contains
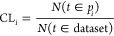
5with *t* corresponding to the targeted heterostructure with defined (CBMDiff, VBMDiff), *p*_*i*_ the *i*th particle constructed from the KNN boosting and associated with DM and CM range values.

For an entire swarm, the total CL score is defined as the ratio of the cumulated targets from the particles p_i_ with the total number of target heterostructures
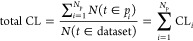
6with CL_*i*_, the CL score associated with the particle *p*_*i*_. It is the CL associated with the swarm pattern obtained. Once information sharing (SL) is performed, the SL score is defined as the ratio of the number of targeted heterostructures to the number of targeted heterostructures in the swarm
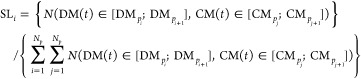
7with SL_*i*_ the SL score of the *i*th secondary particle, DM_*P*_*i*__ the *i*th secondary particle lower DM bound, DM_*P*_*i*_+l_ the *i*th secondary particle upper DM bound, CM_P_ the *j*th secondary particle lower CM bound, and CM_*P*_*i*_+l_ the *j*th secondary particle CM upper bound.

For each particle added to the swarm (increase in swarm size), the SL scores associated with each secondary particle are varying in function of the new borders defined, as a swarm of bees would reorganize its configuration upon the arrival of a new member. From these scores, the best regression results are associated with the secondary particle having the highest total CL and SL values. This secondary particle corresponds to the ranges of DM and CM most associated with the targeted band alignment. To design a heterostructure with targeted band alignment (CBMDiff and VBMDiff as inputs), one should look for samples having DM and CM values included in the ranges delimiting the secondary particle (obtained as outputs) with the best regression results. As it was established earlier that the full 2D/2D heterostructure space cannot be accessed, we use the representative data set collected to evaluate the generalization performance of the model.

### Meta-Estimator Performance and Validation

**Figure 4:**
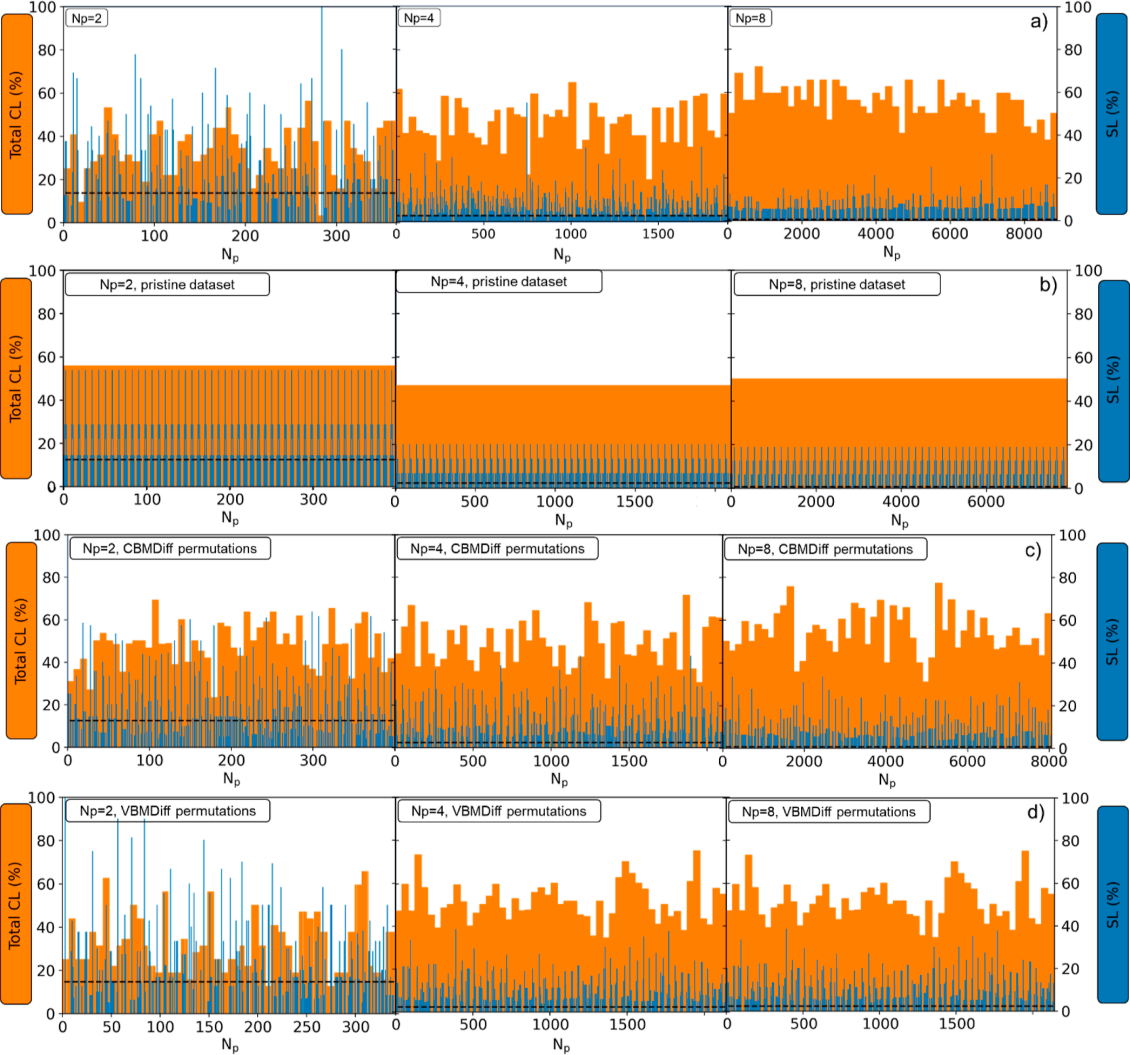
(a) SL (blue) and total CL (orange) scores for a random target value (CBMDiff = 2.8673 ± 0.05 eV and VBMDiff = 1.0441 ± 0.05 eV), (b) SL and total CL scores for the same target value for one iteration of the algorithm with the pristine data set, (c) for a data set with CBMDiff values permuted 50 times, and (d) for a dataset with VBMDiff values permuted 50 times The swarm size (*N*_p_) is increasing from left to right [2 (left), 4 (middle), 8 (right)].

Part of the power of the proposed swarm smart algorithm is that it allows to find small areas of the label space with a high density of heterostructure compounds corresponding to the target. As a consequence, the range of values predicted is smaller, making the meta-estimator more precise. This is shown in Figure 4a, where the SL and total CL scores are presented as a function of the swarm size (*N*_p_) for 50 cumulated runs of the algorithm (Figure 4) of the swarm smart meta-estimator.

The generalization performance of any ML model comes from its ability to predict values with the smallest error possible, i.e., with the highest precision, in testing for descriptor values previously “never seen” by the model. In our case, we test the model for random pairs of CBMDiff and VBMDiff, therefore not corresponding to any sample in the data set. These values are therefore “never seen” by the model, which makes any result a generalization result.

To further validate the model proposed herein, the importance of each feature is described by showing the evolution of SL and CL_T_ scores upon successive permutations of each of the descriptors, as shown in Figure 4c,d. For one iteration of the model (one swarm pattern only), SL and CLT are regularly changing for each permutation of CBMDiff or VBMDiff. This behavior is translating to the prediction of DM and CM being strongly related to the CBMDiff and VBMDiff descriptors through the model. Furthermore, results for permutations of CBMDiff and VBMDiff are different, showing the different importance of these descriptors on the model predictions. These results are valid for the (CBMDiff, VBMDiff) pair chosen in the main text, but also for the different other (CBMDiff, VBMDiff) pairs, as can be seen in Figures S3–S6. The strong relationship is still observed upon modifying the swarm size, and further details are given in the next part regarding the versatility of the model. Hence, the proposed model is valid, and its specificities can be further detailed in the next part.

### Degrees of Freedom

**Figure 5:**
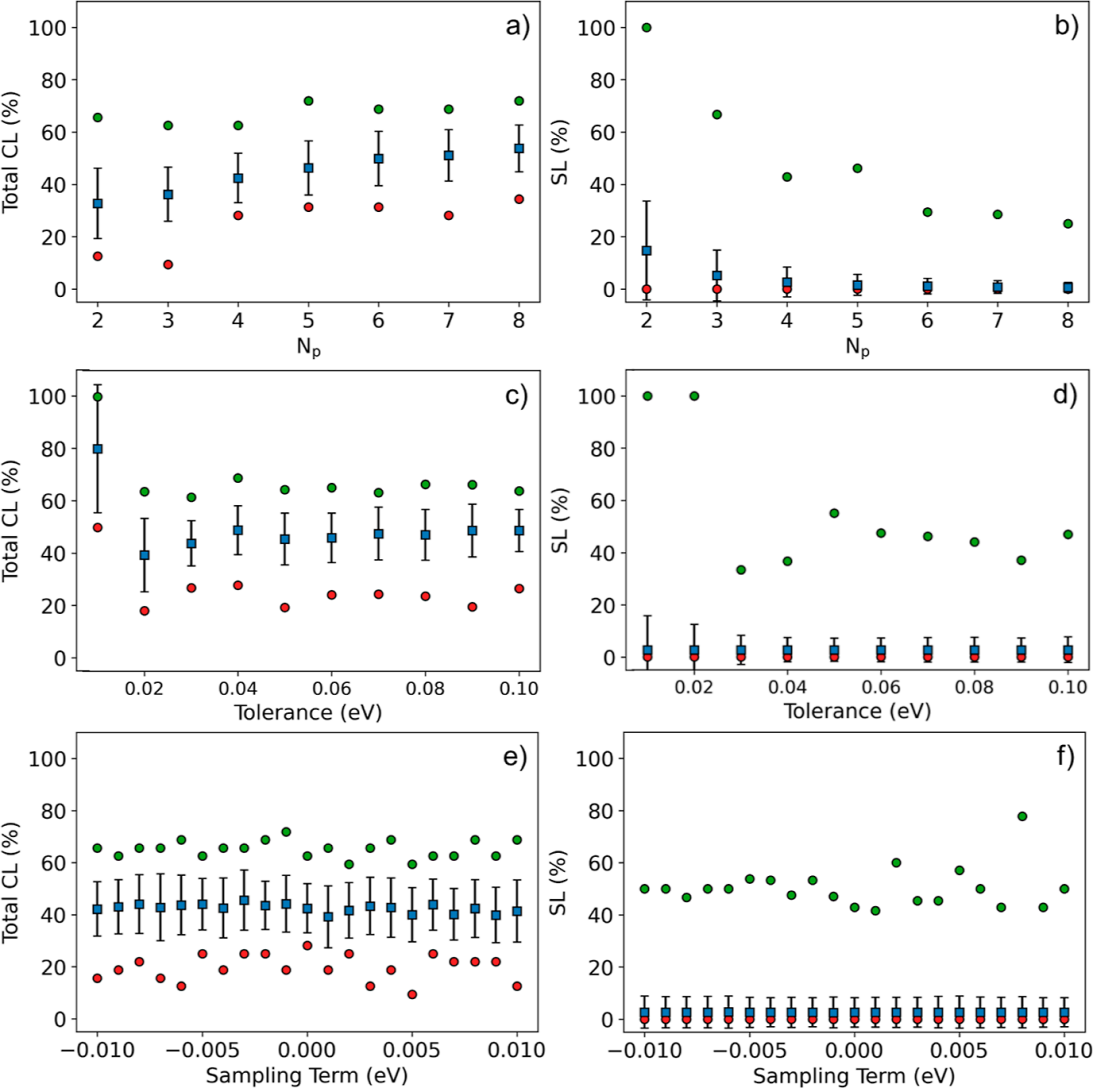
Maximum (green circles), minimum (red circles), average (blue squares) values of (a) SL and (b) total CL scores for a random target value as a function of the swarm size (c,d): maximum (green circles), minimum (red circles), average (blue squares) values of SL (left) and total CL (right) scores for a random target value (CBMDiff = 2.8673 eV, VBMDiff = 1.0441 eV) as a function of the tolerance factor. (e,f): maximum (green circles), minimum (red circles), average (blue squares) values of SL (left) and total CL (right) scores for a random target value as a function of the sampling term. The error bars are computed from 50 iterations of the algorithm.

A first degree of freedom is exhibited here, with the possibility of swarm size modulation. On average, CL_T_ increases with *N*_p_, while the SL score decreases with smaller swarm size while still finding high density regions. Several swarms produce several swarm patterns, which in turn can produce different SL and total CL scores, including the highest ones. Figure 5a,b shows the evolution of the average, minimum, and maximum values of the model as a function of *N*_p_ during the accumulation of 50 runs of the regression algorithm. For CL_T_, the average value is increasing from ca. 30 to 50% with *N*_p_ increasing. The standard deviation is approximately constant throughout the whole swarm size range. This is due to the higher dispersion of CL_T_ values obtained for different regions of the heterostructure space. For SL values, the average is decreasing from 17 to 0% with decreasing *N*_p_ from 2 to 7. Standard deviation is decreasing as well, as *N*_p_ decreases. With regards to the minimum and maximum values, for CL_T_, the maximum is increasing with *N*_p_ from around 60% for *N*_p_ = 2 to around 70% for *N*_p_ = 7. For SL, however, the maximum value almost continuously decreases from 100% (*N*_p_ = 2) to ca. 20% (*N*_p_ = 8). (for the descriptor values randomly picked). The standard deviation decreases with the *N*_p_ as the number of secondary particles increases. This is also explaining the decrease of the maximum SL value, as the secondary particles are becoming smaller with larger *N*_p_ values. This in turn provides regions with low, or even zero, target densities that decrease the average SL value while still providing significantly high target density regions.

The maximum value is always significantly higher than the average for any score and any *N*_p_. This can be explained by the fact that the bigger the swarm, the higher the number of heterostructures, and therefore the higher part of the data set is probed. This is also proof of the model’s ability to single out high density regions in the label space.

Another part of the versatility of the present regression algorithm comes from the built-in tolerance criterion, to control the constraint applied on the regression. Figure 5c,d shows the different scores obtained as a function of chosen tolerance. The SL value is 100% for tolerances of 0.01 and 0.02 eV and then sharply decreases to lower values with fluctuations (for the descriptor values randomly picked). The standard deviation decreases to a constant value from 0.04 eV tolerance. The total CL score is maximum for a tolerance of 0.01 eV but is not significantly higher than the average value. The 0.01 eV tolerance value highlights the importance of this tolerance criterion, as too low a tolerance may constraint the algorithm, that may fail to define a valuable particle despite the SL step. The sharp decrease of total CL and SL scores therefore comes from the transition from a very small amount of targeted descriptor values, where it is easy for the swarm pattern to find all of the desired samples (high CL_T_ and SL scores), to a higher amount of targeted descriptor values (lower CL_T_ and SL scores). The maximum is for CL_T_, and SL scores are also significantly higher than the corresponding average value. The fluctuations of SL and CL_T_ values as a function of tolerance are reflecting the particular distribution of the descriptor values randomly picked.

Yet another aspect of the versatility of the regression algorithm proposed herein is that it can perform for descriptor values in the neighborhood of the nominal value and give different regression results. This sampling term is supposed to be negligible compared with the descriptor value picked, while allowing us to explore a larger portion of the label space. This particularity comes from the data set showing high density while not exhibiting high uniformity; hence, the nearest neighbor search can provide a different result associated with particles that may be located elsewhere, probing another region of the label space. The descriptor value is therefore defined as
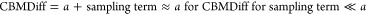
8
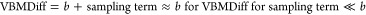
9

As observed in Figure 5c, the maximum total CL fluctuates around 65% for any sampling term, as the minimum total CL shows troughs at less than 20% for sampling terms of −0.009, −0.005, and −0.004 eV. As follows from Figure 5e, the corresponding SL are located around 40% with peaks to 60% for sampling terms of −0.005 and −0.006 eV. The average value is constant and significantly close to its minimum. These results confirm the versatility of the regression found in the use of the sampling term. While all the values shown above correspond to one pair of descriptor (CBMDiff, VBMDiff) values to enable comparison, more descriptor pairs are shown in Figures S5–S8. In these figures, a higher dispersion of CL_T_ values is exhibited as a function of the sampling term, with variations up to around 20% for a 0.001 eV target change. SL is also showing an impact of the change of descriptor values with the sampling term.

The Supporting Information figures include VBMDiff and CBMDiff individual label prediction. While the CL_T_ values are globally lower due to the constraint decrease, SL values show the same trend.

Regarding generalization of the model to heterostructures not included in the data set, as the data set is representative of the 2D/2D heterostructure space, the sample can be generalized to the entire material subspace of 2D/2D heterostructures.^64^ Moreover, labels are neither structure- nor composition-specific, which facilitates comparison between heterostructure samples.

### Relevance of the Model for Material Space Exploration

The heterostructure samples in the data set are a valid illustration of the kind of materials that can be predicted by the model. From the 2Dmatpedia database, 20,164,425 heterostructures can be created. According to extensive literature search performed elsewhere,^36^ only 31 heterostructures have been significantly considered. This corresponds to ca. 0.0001% of the heterostructures that can be created from the 2Dmatpedia database. For the representative data set at hand, those heterostructures correspond to ca. 0.001%; hence, any heterostructure found through using the model is likely not to have been reported in the literature. This shows the strong orientation of the model toward showing genuinely new samples, steering the 2D/2D heterostructure discovery process. Nevertheless, few samples from the data set used here are representative of the type of combinations that the algorithm can find.

Table 1 exhibits a nonexhaustive, although diverse, list of such heterostructures. Associated to these samples, application examples are given to ensure that the predictions can have an impact on different fields of applied sciences.

**Table 1:** Different Heterostructure Samples from the Dataset Also Found in Literature^a^

heterostructure	study type	applications	refs
Sn_2_S/Bi_2_Se_3_	experimental	photodetection	(65)
SnS/SnS_2_	experimental	lithium-sulfides batteries	(66)
C_3_N_4_/SnS_2_	experimental	hydrogen production	(67)
Nb_2_O_5_/C_3_N_4_	experimental	hydrogen production	(68)
PbTe/SnTe	experimental	topological insulators	(69)
In_2_S_3_/SnS_2_	experimental	organic pollutant removal	(70)
ZrS_2_/HfS_2_	computational	optoelectronics, nanoelectronics	(71)
C_3_N_4_/ZrS_2_	computational	photocatalysis	(56),^57^
SnO_2_/C_3_N_4_	_	organic pollutant removal	(72),^74^
MoO_3_/C_3_N_4_	_	organic pollutant removal	(73),^75^

a The first column is showing the heterostructure composition, the second column indicates the type of study reported in the literature, and the third column gives the reference from the literature. “_” entries correspond to prospective heterostructure samples, not yet studied despite their availability as separated materials.

Experimentally known heterostructures were found,^62−71^ that are used for different applications ranging from photodetection to batteries, including photocatalysis. Heterostructure specimens can also be found only computationally,^56,57,71^ considered for nanoelectronics or photocatalytic goals. The model can also predict heterostructures that have not yet been considered, either experimentally or computationally. Those can still exist as readily synthesizable 2D materials^72,73^ with prospective applications^74,75^ and are prospective new heterostructure samples. This last part shows that the model can also steer heterostructure research toward new horizons by providing motivation to study previously overlooked compounds.

## Conclusions

This work is proposing a new way to choose candidates for 2D/2D heterostructure fabrication, either from two new materials or from one host. Starting from two general and easily computable labels (DM and CM), a 2D label space is created. Those labels alleviate the limitations of other conventional heterojunction characteristics (LM), along with keeping a low-dimensional label space. While helping in singling out heterostructure samples with a given band alignment to look for correlation among other specific properties, it can be further improved by including correction to Anderson’s rule recently reported^76^ or even by modifying the base model using RF or support vector regression for example. The meta-estimator developed here can be readily trained on other systems to bolster compound selection either on other systems (e.g., 0D/2D, 1D/2D heterostructures) or other fields (e.g., photocatalysis).

## Data Availability

The data and code used for this study are available at: 10.5281/zenodo.8248512.

## Supporting Information Available

Scoring results of 50 runs of the algorithm and 50 permutations of the labels for partial band alignment with varied degrees of freedom, and scoring results of 50 runs of the algorithm and 50 permutations of the labels for total and partial band alignment with varied degrees of freedom with two random pairs (PDF)
