# Supplementary material for: Swarm Smart Meta-Estimator for 2D/2D Heterostructure Design
Source: J Chem Inf Model. 2023 Oct 5;63(20):6212–23. doi: 10.1021/acs.jcim.3c01509 (PMC10598791; doi:10.1021/acs.jcim.3c01509)
Supplement: Supplementary file 1 — ci3c01509_si_001.pdf [file ci3c01509_si_001.pdf]

Supporting Information

Swarm smart meta-estimator for 2D/2D heterostructure design

Romain Botella, Andrey A. Kistanov, Wei Cao*

Nano and Molecular Systems Research Unit, University of Oulu, Oulu 90014, Finland

Corresponding author: Romain.Botella@oulu.fi

In the following curves, the reduced Mean Absolute Deviation is used:

$$reduced\ MAD = \frac{MAD}{Max(descriptor) - Min(descriptor)}$$

for any descriptor

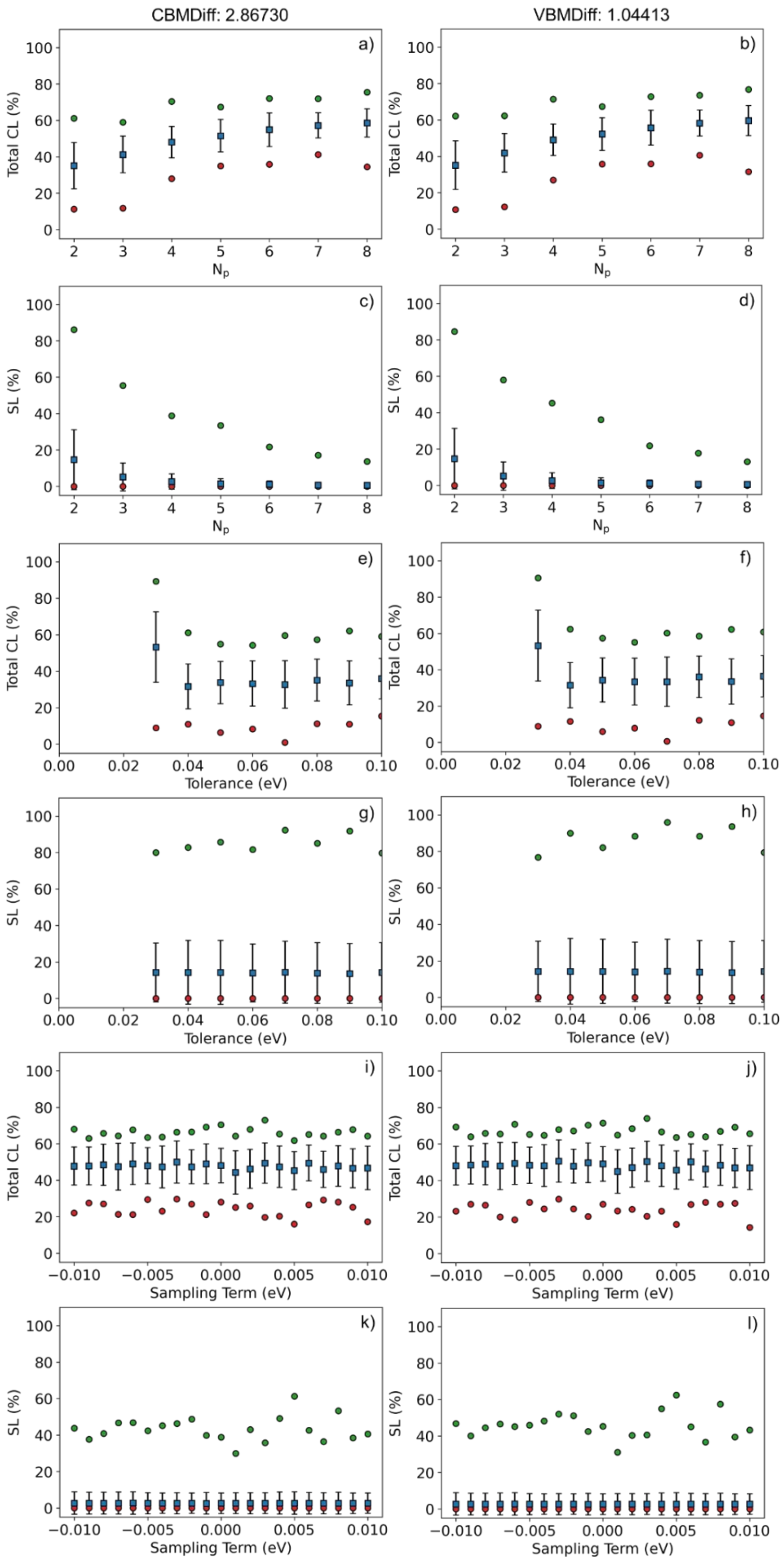

Fig. S1 Evolution of Total CL and SL scores for partial band alignment prediction (CBMDiff = 2.86730 ± 0.05 or VBMDiff = 1.04413 ± 0.05) as a function of a) to d) swarm size, e) to h) Tolerance and i) to l) Sampling Term. green circles, red circles and blue squares correspond to the maximum value, minimum value and average value out of 50 runs of the algorithm, respectively.

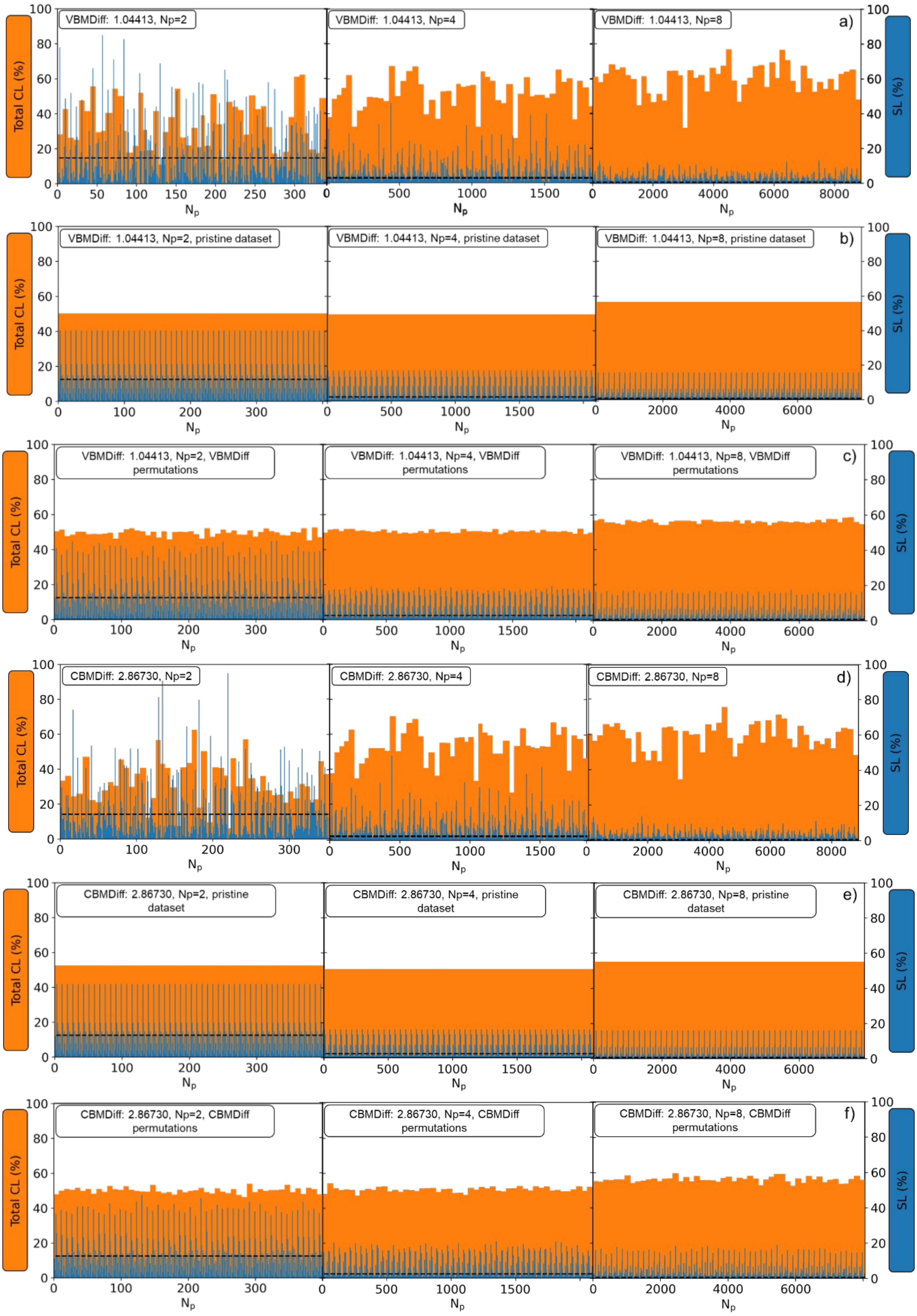

Fig. S2. Superposed evolution of Total CL (orange) and SL (blue) scores for partial band alignment (CBMDiff = 2.86730 ± 0.05 or VBMDiff = 1.04413 ± 0.05) for a) and d) 50 runs of the algorithm, c) and f) 1 run of the algorithm (1 swarm pattern) and 50 permutations of CBMDiff values (f) and VBMDiff values (c) and b) and

e) control runs (1 swarm pattern and no permutations) for different swarm sizes: 2 (left), 4 (center) and 8 (right). Black dashed lines are average values of SL.

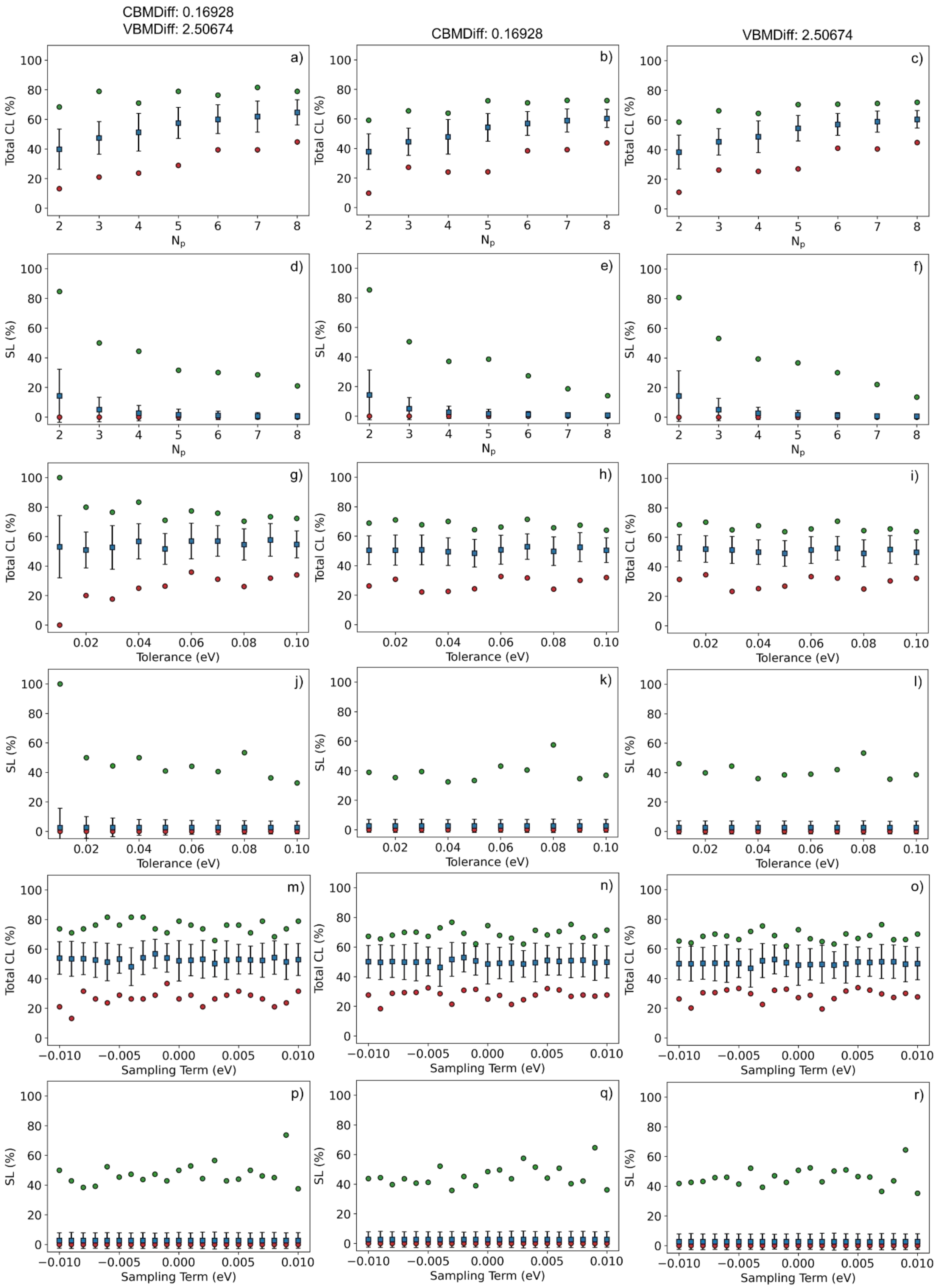

Fig. S3. Evolution of Total CL and SL scores for total (CBMDiff = 0.16928 ± 0.05 and VBMDiff = 2.50674 ±

0.05) and partial band alignment prediction (CBMDiff = 0.16928 ± 0.05 or VBMDiff = 2.50674 ± 0.05) as a function of a) to f) swarm size, g) to l) Tolerance and m) to r) Sampling Term. green circles, red circles and

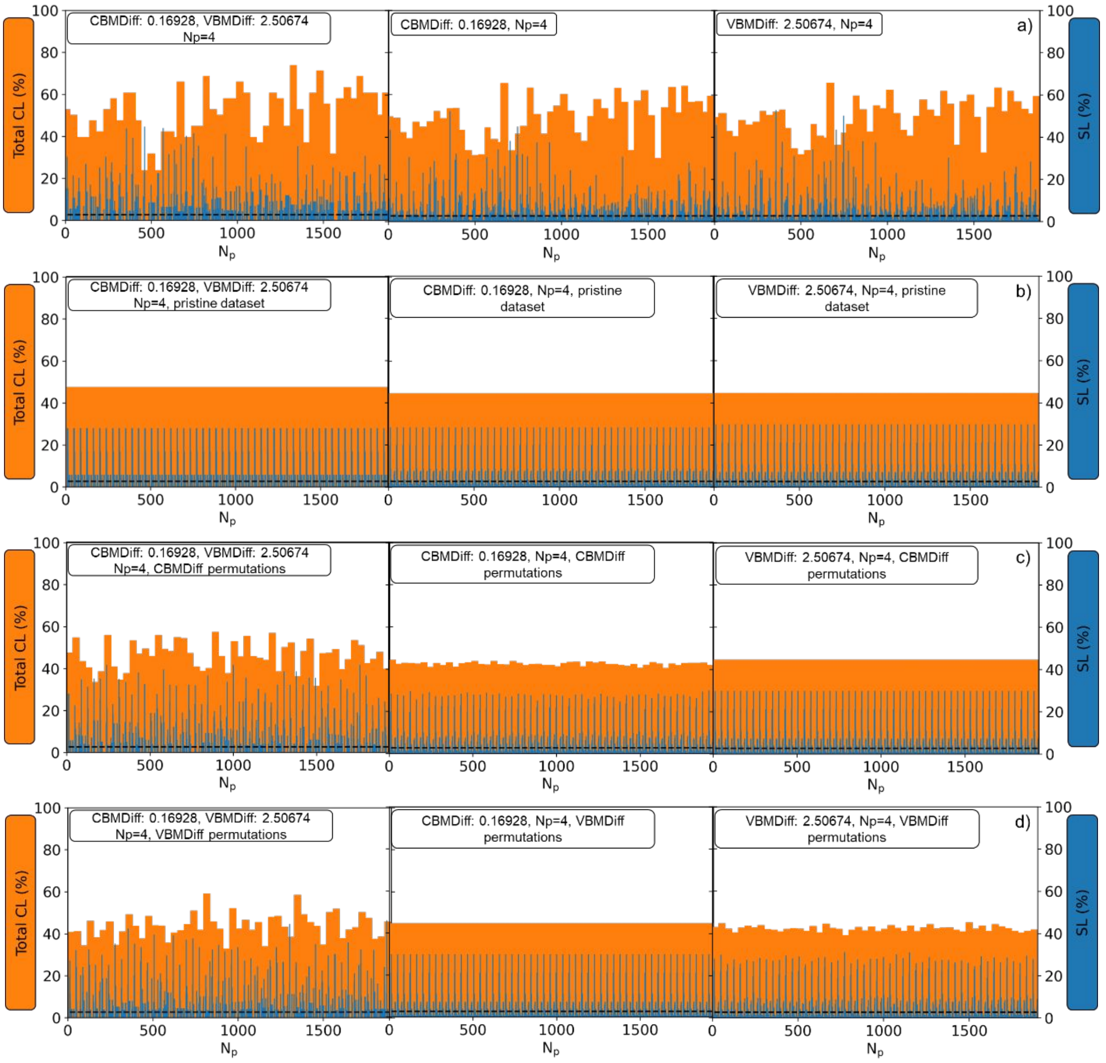

blue squares correspond to the maximum value, minimum value and average value out of 50 runs of the algorithm, respectively.

Fig. S4. Superposed evolution of Total CL (orange) and SL (blue) scores for total (CBMDiff = 2.50674 ± 0.05 and VBMDiff = 0.16928 ± 0.05, left side) and partial band alignment (CBMDiff = 2.50674 ± 0.05, center or VBMDiff = 0.16928 ± 0.05, left side) for a) 50 runs of the algorithm, b) control runs (1 swarm pattern and no permutations), and 1 run of the algorithm (1 swarm pattern) and 50 permutations of c) CBMDiff values and d) VBMDiff values. Black dashed lines are average values of SL.

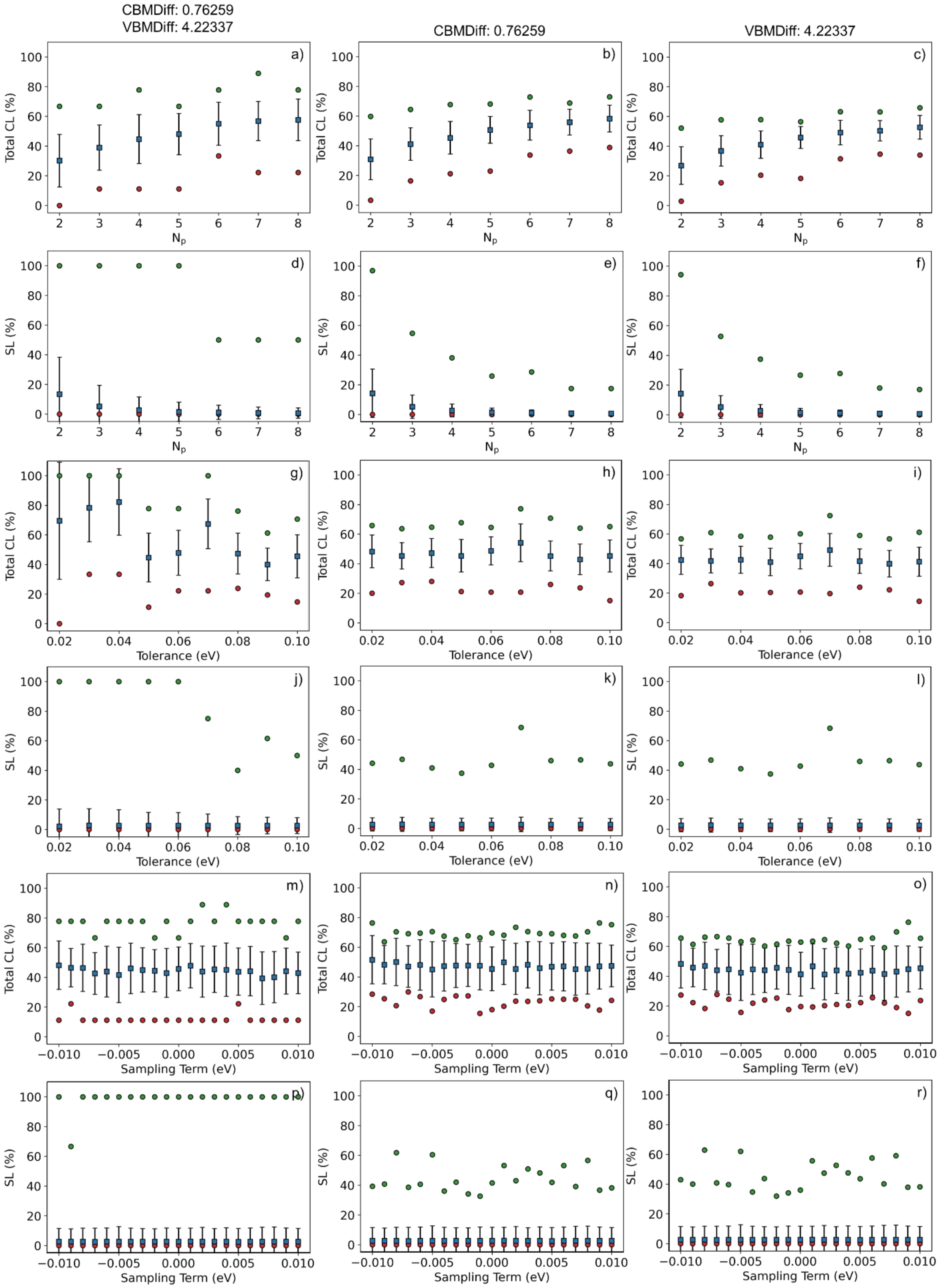

Fig. S5. Evolution of Total CL and SL scores for total (CBMDiff = 0.76259 ± 0.05 and VBMDiff = 4.22337 ±

0.05) and partial band alignment prediction (CBMDiff = 0.76259 ± 0.05 or VBMDiff = 4.22337 ± 0.05) as a function of a) to f) swarm size, g) to l) Tolerance and m) to r) Sampling Term. green circles, red circles and blue squares correspond to the maximum value, minimum value and average value out of 50 runs of the

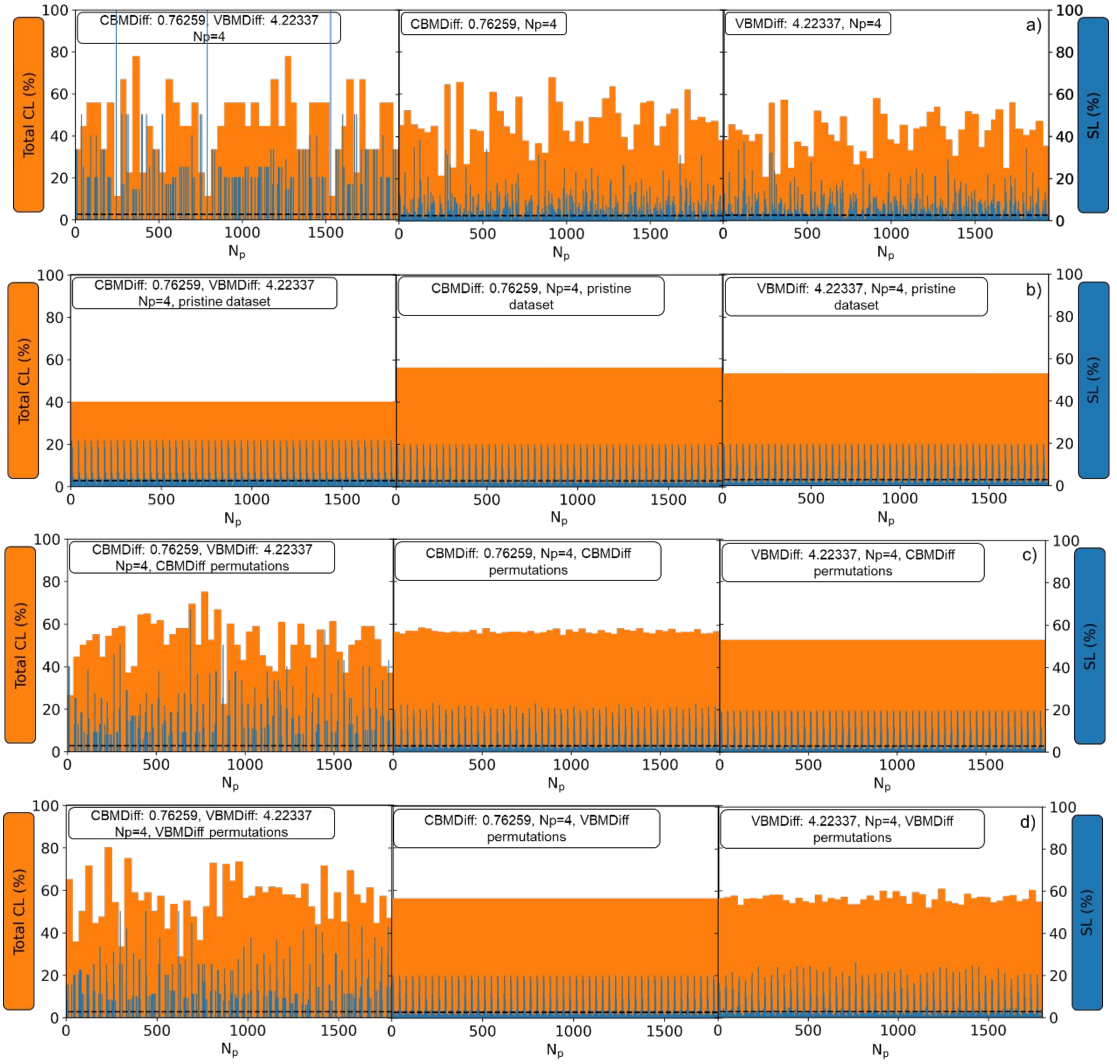

algorithm, respectively.

Fig. S6 Superposed evolution of Total CL (orange) and SL (blue) scores for total (CBMDiff = 0.76259 ± 0.05 and VBMDiff = 4.22337 ± 0.05, left side) and partial band alignment (CBMDiff = 0.76259 ± 0.05, center or VBMDiff = 4.22337 ± 0.05, left side) for a) 50 runs of the algorithm, b) control runs (1 swarm pattern and no permutations), and 1 run of the algorithm (1 swarm pattern) and 50 permutations of c) CBMDiff values and d) VBMDiff values. Black dashed lines are average values of SL.
